# Special Issue “New Perspective in the Molecular Pathways Involved in Acute and Chronic Lung Injury”

**DOI:** 10.3390/ijms27010080

**Published:** 2025-12-21

**Authors:** Marika Cordaro

**Affiliations:** Department of Biomedical, Dental and Morphological and Functional Imaging, University of Messina, 98125 Messina, Italy; marika.cordaro@unime.it

Acute and Chronic Lung Injury (ALI and CLI) represents a growing global health threat [1]. Through the coupling of a constantly increasing world population and a lifestyle characterized by exposure to environmental pollutants, the World Health Organization (WHO) estimates that 1 billion individuals will suffer from acute and/or chronic lung diseases in the next 10 years due to air pollution exposure, infection (viruses, bacteria, and other pathogenic microorganisms), and specific genetic factors [2,3]. The lungs, being one of the few internal organs constantly exposed to a wide range of exogenous organic, inorganic, and biological agents, are, as a consequence, particularly vulnerable to the development of simple-to-complex disorders, which can compromise quality of life and, ultimately, even lead to death [4]. Severe conditions such as acute respiratory distress syndrome (ARDS) and idiopathic pulmonary fibrosis (IPF) are the culmination of a complex cascade involving multiple mechanisms such as barrier disruption, dysregulated inflammation, and aberrant tissue repair [5,6]. The current therapeutic approach includes bronchodilators, corticosteroids, and antibiotics [7], which only target the primary symptoms of the disease, without providing any long-term relief, neither in terms of prevention nor in terms of cure [8,9,10,11].

To overcome this limitation, the key lies in identifying and targeting the underlying pathways that drive tissue damage. The following Special Issue, entitled “New Perspectives in the Molecular Pathways Involved in Acute and Chronic Lung Injury”, includes five fundamental scientific contributions that offer new molecular strategies and therapeutic perspectives.

Disruptions in endothelial and epithelial barriers play a key role in the pathogenesis of ALI and ARDS. Barrier dysfunction facilitates the dysregulated trafficking of immune cells into tissues, resulting in chronic inflammation and tissue damage [12,13].

The authors of two scientific contributions included in this Special Issue explore the underlying mechanisms, one focusing on intrinsic cell biology and the other on the impacts of external agents.

The findings of the study by Adil et al. (2025) [14] demonstrate the protective function of the tight junction protein Claudin-17 (Cldn17). Cldn17 is established as a critical regulator of vascular permeability and immune homeostasis. The results show that the absence of this protein in mouse models leads to a significant increase in vascular permeability and the development of basal pulmonary edema. Its deficiency drives vascular leakage, exacerbates lung injury, and alters immune signaling pathways, underscoring its potential as a therapeutic target for inflammatory lung diseases.

In the article by Hamon et al. (2024) [15], the authors investigate how exogenous pollutants compromise the first line of defense. The authors report that dual exposure to e-cigarette vapor and cigarette smoke leads to worse functional outcomes in cells compared to single exposure to either type alone. This combination leads to an increase in toxicity, reduced metabolism in airway epithelial cells, reduced monocyte migration, macrophage phagocytosis, and altered chemokine production. Both cigarette smoke and e-cigarette vapor are known to contain highly reactive compounds [16,17,18,19,20], which, upon combination, could easily form unique chemical adducts that dissolve into the fluid of the lung luminal space, leading to the activation of entirely new molecular pathways. It can be concluded that dual use exposes the user to an increased number of chemicals compared to smoking alone.

One of the current gaps in lung therapy is the lack of effective treatments to prevent or reverse progression from the acute inflammatory phase to chronic fibrotic remodeling. The authors of two of the included articles explore novel strategies in this direction, focusing on the key pathways of inflammation and extracellular matrix (ECM) deposition.

The findings presented in the article by Paik et al. (2024) [21] offer a mechanistic validation for a drug already approved as an antifibrotic, Pirfenidone. The results showed that pirfenidone administration suppressed inflammation and fibrosis in the ARDS animal model. As a result, pirfenidone can be considered as a new early treatment option for ARDS. Treatment with pirfenidone strongly suppressed the expression of TGF-β1/Smad-2 signaling factors and improved the imbalance of MMP-9/TIMP-1. These effects led to a decrease in levels of fibrosis factors and pro-inflammatory cytokines, promoting the recovery of damaged lung tissue. It can thus be concluded that the modulation of the TGF-β1/Smad signaling pathway is vital in improving lung fibrosis in ARDS, as it is involved in inflammation, mesenchymal transition, and ECM deposition [22,23]. Moreover, in patients with ARDS, the excessive production of MMP-9 can destroy the basement membrane, enable fibroblasts to invade the alveolar space, and lead to pulmonary fibrosis [24,25]. MMP-9 is constitutively suppressed by TIMP-1, such that an imbalance between MMP-9 and TIMP-1 plays a role in the pathogenesis of ARDS [24]. Pirfenidone inhibits the expression of MMP-9 and TIMP1 directly or by reducing the synthesis of TGF-β and downstream mediators, and it may also reduce TGF-β activation by MMPs [26].

A radically new treatment approach is proposed by Genovese et al. (2023) [27]. The study findings clearly provide the first evidence that the inhibition of FAAH (fatty acid amide hydrolase) activity may represent a potential therapeutic strategy. FAAH inhibition increases endogenous levels of endocannabinoids, which exert a protective effect that manifests itself through decreased NF-kb translocation and, as a consequence, the release of TNF-α, IL-1, and IL-6. The results of this research strengthen our understanding of how FAAH modulates inflammation pathophysiologically and lends credence to its therapeutic potential for chronic lung illnesses. This discovery opens the door to therapies based on the endocannabinoid system for the management of IPF.

Lastly, in the review by Mokra et al. (2025) [28], the authors provide a critical assessment of a therapeutic strategy based on anti-oxidation. N-acetylcysteine (NAC) is a widely used therapeutic agent because of its potent antioxidant and mucolytic action [29,30,31]. However, NAC also possesses numerous anti-inflammatory, anti-fibrotic, and cytoprotective effects, which support its use in the treatment of various inflammatory disorders, including pulmonary diseases [32]. The advantages of using NAC are mainly its low cost, wide availability, low toxicity, and few side effects. The review authors note that early administration of NAC may reduce markers of oxidative stress and alleviate inflammation in animal models of acute lung injury (ALI) and in patients suffering from distinct forms of acute respiratory distress syndrome (ARDS) or pulmonary infections, including community-acquired pneumonia or Coronavirus Disease (COVID)-19. The authors report that in spite of favorable findings from animal studies, the results of clinical trials are rather heterogeneous. The discrepancy likely results from heterogeneity in dosage and the NAC administration mechanism, the inclusion/exclusion criteria of the trials, the composition of patient groups, the subtype of ARDS, etc. The authors conclude that although NAC is relatively safe, cost-effective, and widely available, further research is required, with a particular focus on its combination with other therapies and the development of drug delivery strategies, such as nanocarriers, to overcome bioavailability limitations.

The papers presented in this Special Issue chart a clear path for future research on lung injury. The contributions demonstrate that the therapeutic approach must not be monolithic but instead consider the specificity of the damage and the phase of the disease. The results of studies on the role of pathways such as NF-kb (involved in FAAH and NAC), TGF-β\Smad, and structural integrity (Cldn17) suggest that the identification of pathogenic subtypes and the targeted administration of specific therapies for each molecular pathway represent the true “New Perspective” in the fight against lung injury.
